# Rapid Sensing of Biological and Environmental Analytes Using Microwave-Accelerated Bioassays and a MATLAB Application

**DOI:** 10.5101/nbe.v11i2.p111-123

**Published:** 2019-04-15

**Authors:** Enock Bonyi, Edward Constance, Zeenat Kukoyi, Sanjeeda Jafar, Kadir Aslan

**Affiliations:** 1 Department of Civil Engineering, Morgan State University, 1700 East Cold Spring Lane, Baltimore, Maryland 21251, USA.; 2 Department of Biology, Morgan State University, 1700 East Cold Spring Lane, Baltimore, Maryland 21251, USA.

**Keywords:** Analyte, Colorimetric bioassays, Microwave-accelerated bioassays, Microwave heating, MATLAB

## Abstract

We report a method for rapid detection and analysis of biological and environmental analytes by microwave-accelerated bioassays (MABs) and a novel MATLAB-based image processing of colorimetric signals. In this regard, colorimetric bioassays for histidine-rich protein 2 (HRP-2) and microcystin-leucine arginine (MC-LR) toxin were carried out using MABs and without microwave heating (i.e, gold standard bioassays). Our MATLAB-based detection method is based on the direct correlation of color intensity of a solution calculated from images captured with a smartphone with the concentration of the biomolecule of interest using a MATLAB code developed in-house. We demonstrated that our MATLAB-based detection method can yield bioassay sensitivity comparable to the colorimetric gold standard tool, i.e., UV-Visible spectroscopy. In addition, colorimetric bioassay time for the HRP-2 assay (used in malaria diagnosis) and colorimetric MC-LR bioassay (used in MCLR toxin diagnosis) was reduced from up to 2 hours at room temperature without microwave heating to 15 minutes using the MABs technique.

## Introduction

Detection and quantification of analytes related to human health in biological and environmental samples have been the primary focus of scientists, health providers, and policy-makers for several decades. In this regard, conventional methods, such as, colorimetric bioassays, blood cell analyzers and polymerase chain reaction (PCR) are routinely used in analysis of biolological samples [1]. In addition, culture-based techniques for microbiological analysis, biochemical identification methods are used as confirmatory tests for samples that test positive in spectroscopic and chromotographic methods (e.g., mass spectroscopy, gas chromatography, high-performance liquid chromatography, and intact cell mass spectrometry [2–5]).

While the methods mentioned above are widely used, they can lack sensitivity, specificity, portability, rapidity, repeatability, and can have high operational and materials costs. Furthermore, most of these methods can require large quantities of sample, highly trained and certified personnel. In that regard, there has been a continuous drive to develop new technologies and tools that can overcome these limitations of the current methods and instrumentation.

Recently, the Aslan Research Group has demonstrated that metal nanoparticles, such as, silver island films impregnated on traditional platforms (high throughput screening wells and glass substrates), improved bioassay sensitivities and significantly reduced bioassay time when coupled with low power microwave heating [6, 7]. Other researchers have developed technologies, such as, immunomagnetic electroluminescence (ECL) [8], microwave-accelerated metal-enhanced fluorescence (MAMEF) [7, 9–11], aptamer-magnetic bead ECL, biosensors, which have shown to improve the sensitivity of bioassays. However, these methods still require the use of traditional spectroscopy tools for optical measurements. New point-of-care tools for medical diagnostics and on-site monitoring of environmental samples necessitate the development of tools and software that can be employed at locations away from the typical laboratory setting.

In this work, we report a new method to analyze biological and environmental samples and the technique involves the use of image processing of colorimetric signals (using a MATLAB-based application) collected from two bioassays (HRP-2 and MC-LR toxin) completed on hybrid platforms using MABs technique and at room temperature (no microwave heating, control experiments). Our MATLAB-based detection method has yielded comparable bioassay sensitivity to the standard method (i.e., ultraviolet–visible spectroscopy (UV-Vis)) and reduced the colorimetric bioassay time for HRP-2 assay (used in malaria diagnosis) and colorimetric MC-LR bioassay (used in MC-LR toxin diagnosis). Our approach has afforded accurate detection of colorimetric signals using a smartphone, therefore, proven to be a potential tool for in situ detection of biological and environmental analytes by providing an inexpensive alternative to the current instrumentation.

## Experimental

### Materials

#### Chemicals and reagents

Poly(methyl methacrylate) (PMMA) discs (diameter = 5 cm, thickness = 0.2 cm) were acquired from McMaster-Carr (Robbinsville, NJ, USA), 96-well high throughput plates were purchased from Sarstedt (Newton, NC, USA) and indium tin oxide (ITO)-coated plastic PET films (height = 0.175 mm, width = 300 mm, length = 1 m, resistivity = 14 Ω/sq), were purchased from MTI Corporation (Richmond, CA, USA). Deionized water with 18.2 mΩ.cm resistivity at 25 °C was achieved using Millipore Direct-Q UV3 system with a 0.22 μm filter. Copy paper (8.5” × 11”) was bought from Staples (Towson, MD, USA), phosphate buffered saline (PBS) pellets, and sodium phosphate-citric buffer was prepared using deionized water.

#### Proteins and antibodies

Bovine serum albumin (BSA), streptavidinconjugated horseradish peroxidase (strep-HRP), biotinylated bovine serum albumin (b-BSA), substrate o-phenylenediamine dihydrochloride (OPD) and protein A were bought from Sigma-Aldrich Corp. (Milwaukee, WI, USA). Recombinant falciparum histidine-rich protein-2 (HRP-2) was bought from Bio-Rad (Raleigh, NC, USA). Mouse monoclonal antibody against HRP-2 and ELISA kit for HRP-2 assay were obtained from Abcam (Cambridge, MA, USA). Whole blood was acquired from Good Samaritan Hospital (Baltimore, MD, USA). ELISA kit for MC-LR was purchased from Abnova (Walnut, CA, USA).

#### Instrumentation

Microwave heating was performed using a 700 W Emerson microwave and an in-house built 100 W iCrystal microwave system. Absorbance values were measured using Agilent UV-Vis spectrophotometer (Santa Clara, CA, USA). SigmaPlot version 12.5 was used for statistical analysis of all data. ITO circles were obtained from ITO sheet using a 1-hole punch and uniform lighting on HTS wells was achieved through ultra-thin LED light box; all were acquired from Staples (Towson, MD, USA). Pictures of the bioassays were taken using a 12 MP iPhone smartphone camera (f/2.2 aperture, five-element lens, noise reduction and IR filter). Harrick plasma cleaner used to clean the ITO surfaces was purchased from Harrick Plasma Inc. (Ithaca, NY, USA). Our MATLAB-based application was installed and run on HP computer running on Windows 10.

### Methods

#### Development of a MATLAB application

The Matlab-enabled application was developed using MATLAB language licensed under MathWorks. Fig. S1 (Supp. Information) shows the layout of the novel MATLAB application with four sections: image upload area section, graphical and histogram output section, tabular output section and settings section. The image upload area section provides space to display the sample images whereas the graphical, tabular and histogram output section displays the pixel output for each sample and provides a plot for each concentration.

#### Pixel Normalization

Equation (1) was used to perform pixel normalization on experimental and standard samples pixel output.

Normalized sample pixels (Np) = Sample pixels (Sp)/ Blank well pixels (Bwp)

(1) Np=(Sp/Bwp),

where, Sp represents grayscale and RGB pixels in the range 0–255 for experimental and standard samples, and Bwp represents grayscale and RGB pixels in the range 0–255 for blank well samples.

#### Evaluation of MATLAB application under different light environments using model b-BSA bioassay

Protein assay, b-BSA (a model protein) was carried out as described in our previous work [12, 13]. The colorimetric detection method was used to report the magnitude of the enzymatic signal. After the development of the colored product and the reaction was stopped with a stopping solution, optical images of the sample wells were taken using an iPhone smartphone camera (12 MP) under the following environments; (i) on the laboratory bench with incident light (room light) and light emitting diode (LED) light source lighting the backside of the HTS wells, (ii) in a dark room with only the LED light source lighting the backside of the HTS wells. [Note: HTS wells were painted black on the sides of the wells, and the bottom side left clear to avoid cross-talk between the wells]. Optical images were saved using JPEG file format and later uploaded into the GUI window of the main program using the insert button. Once the parameters (i.e., the number of rows, the number of columns, diameter range, and pixel range) were set, pixel computation in each well was done by pressing the run button and this yielded a graphical and tabular output.

#### Preparation of real-life bioassays: HRP-2 and MC-LR bioassays

##### Collection, processing and storage of blood and its components

Blood samples with purple caps and without patient identifiers were obtained from the Good Samaritan Hospital located in Baltimore, MD, USA. It is important to note that blood samples were specimens drawn by hospital phlebotomists via venipuncture for patient diagnostics and were destined for disposal after seven days as per the hospital policy. After the expiry of the policy timeline, the blood samples were transported to our laboratory at Morgan State University in an insulated ice bucket according to IRB approved procedures. Upon receipt, the samples were centrifuged at 2500 rpm for 10 minutes using Eppendorf 5810R centrifuge and the supernatant, serum, aspirated using a pipette into plastic screwcapped vials. The remaining blood samples were kept at +4 °C. The serum was aliquoted and kept in Ziploc bags labeled bio-hazard and frozen at -82 °C until when needed for experiments.

##### Dilution of monoclonal antibodies against HRP-2 in buffer (PBS), serum, and blood

The frozen serum and refrigerated blood were left to thaw and warm to room temperature. Mouse monoclonal antibodies against HRP-2 and MC-LR were diluted in PBS in ratios of 1:50, 1:100, 1:500, 1:1000, 1:5000, and 1:10000. To mimic the human body environment, the same setup was repeated except that the mouse monoclonal antibodies were diluted in whole blood and serum. Antibodies were stored at +4 °C.

##### Preparation of HRP-2 and MC-LR antigens

A stock solution of HRP-2 antigens received from the manufacturer was diluted ten-fold using PBS (pH = 7.4) and stored at +4 °C. MC-LR antigens were used as received (i.e., 0, 0.5, 1.0, 2.0, and 2.5 μg/L).

##### Analysis of indirect and competitive bioassay for HRP-2 and MC-LR on ITO using our software

**Scheme 1:**
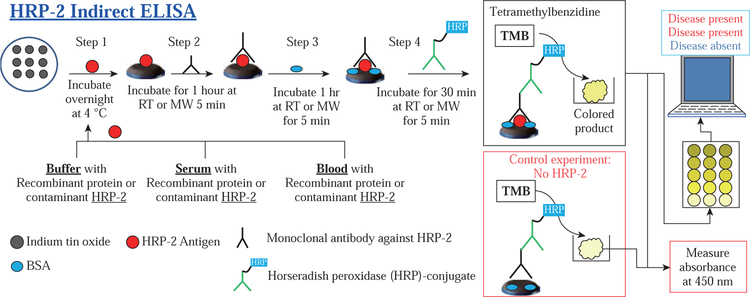
Schematic depiction of indirect bioassay for HRP-2 in blood, serum and buffer performed on ITO bioassay platforms at room temperature and using the MABs technique. Total assay time after step 1: Room temperature (RT) without microwave heating = 125 mins, and the MABs technique with microwave heating (MW) < 20 mins.

**Scheme 2:**
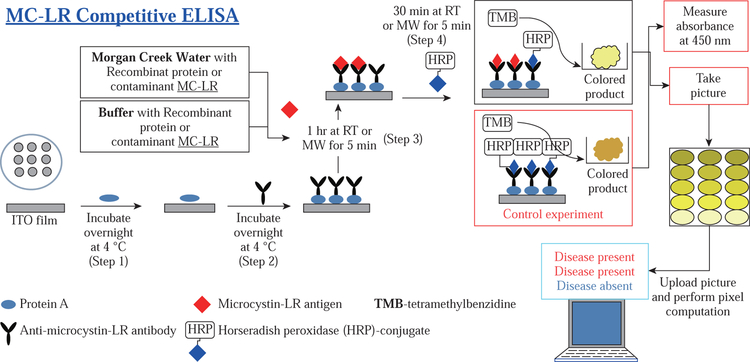
Schematic depiction of a competitive bioassay for microcystin-LR (MC-LR) in MSU creek water and in buffer and performed on ITO at room temperature and under microwave heating (MABs technique). Total assay time after step 2: Room temperature (RT) without microwave heating = 105 minutes, and the MABs technique with microwave heating (MW) < 20 minutes.

Schemes 1 and 2 show the schematic representation for the indirect HRP-2 and MC-LR assays performed using the MABs technique and at room temperature (no microwave heating as a control experiment), respectively. In HRP-2 bioassay, the HRP-2 antigens were incubated overnight at +4 °C on ITO bioassay platforms. Unbound antigens were washed off using PBS wash buffer. Mouse monoclonal antibodies against HRP-2 were added and microwave heated for 5 minutes or incubated for 1 hour at room temperature. After washing the wells three times with washing buffer, detection antibody, goat anti-mouse conjugated to HRP was added to the wells and microwave heated for 5 minutes or incubated for 1 hour at room temperature. A colorimetric bioassay substrate (i.e., tetramethylbenzidine, TMB) was added and left to incubate at room temperature for 15 minutes or microwave heated for 5 minutes. The well contents were transferred into the HTS wells with outer-walls painted black and containing stop solution of sulfuric acid. An iPhone 7 smartphone camera was used to take optical images of the colored product inside the wells. Pixel count on the picture images was performed using the novel MATLAB-based detection application developed in-house.

For the MC-LR bioassay, ITO bioassay platforms were coated with protein A and incubated overnight at +4 °C (Fig 2, step 1). After washing the excess protein A from the wells, mouse monoclonal antibodies were introduced into the wells and left to incubate overnight at +4 °C (Scheme 2, step 2). HRP-2 antigens of different concentrations noted in section 2.2.3.2 were added to the wells and incubated at room temperature for one hour or microwave heated for 5 minutes after which HRP-conjugate was added to the wells and microwave heated for 5 minutes or incubated at room temperature without microwave heating for 30 minutes. Unbound conjugate and MC-LR antigen were washedoff the wells, and TMB substrate added. Bioassay platform was microwave heated for 5 minutes or left at room temperature without microwave heating for 15 minutes to allow color development for the bioassay. Real color images and pixel computation for MC-LR assay was done using a 12 MP iPhone smartphone camera and MATLAB-based application as previously described under the HRP-2 assay.

##### Analysis of indirect and competitive assay for HRP-2 and MC-LR using commercial immunoassay kits and UV-Vis spectrophotometer

Each assay for HRP-2 and MC-LR was performed as per the manufacturer’s recommendations. In brief, the kits and its contents were left to attain room temperature. HTS wells were already pre-coated with corresponding antigens for HRP-2 and antibodies against MC-LR for HRP-2 and MC-LR bioassays, respectively. After which the assaying process progressed as described in the previous section with HRP-2 assay starting after step 1 (Scheme 1) and MCLR after step 3 (Scheme 2). Absorbance measurements of the colored product were carried out at 450 nm using an UV-visible spectrophotometer.

#### Data analysis

Data generated from UV-visible spectrophotometer and our new MATLAB software were stored in Microsoft Excel files and analyzed using SigmaPlot version 12.5.

### Results and Discussion

#### Development and testing of the efficacy of the MATLAB application

To assess the efficacy of the MATLAB application to convert color intensity into numerical grayscale values, we generated a gradient (shades) of yellow color using the different volumes of a colorimetric bioassay substrate (OPD) and constant volume of HRP (enzyme) at room temperature without microwave heating. Fig. S2 (Supporting Information) shows normalized pixels (obtained using Eq. (1)) for images of colored enzymatic product taken under four different environments: (1) under direct sunlight on LED board (solid black line with filled circles), (2) under direct sunlight on LED board with a shade (red solid line with unfilled circles), (3) under direct sunlight on a white paper (green line with filled inverted triangles) and (4) under direct sunlight on a white paper with a shade (blue solid line with unfilled upright triangles). From these normalized pixel results, it was also evident that there was minimal variation in pixels for sample images taken under a shade (blue and solid red lines) compared to those acquired in direct sunlight (black and green solid lines), which implies that normalization of pixels generated from bioassay images taken in different is important for consistent and accurate results.

#### Comparison of performance between MATLAB application and UV-Vis spectrophotometry for real-life HRP-2 assay sensing

**Fig. 1:**
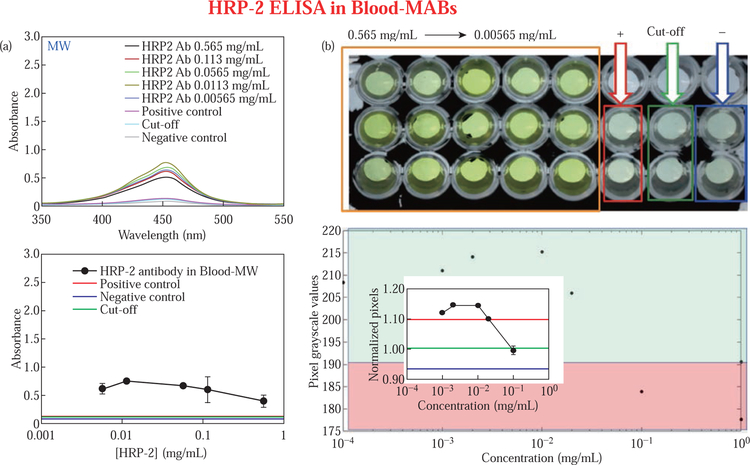
Absorption spectra for HRP-2 assay in blood performed on ITO bioassay platform **(a)** using the MABs technique and normalized grayscale pixel values computed using the new diagnostic software (**(b)**, bottom panel). The real-color pictures of experimental samples (solid orange enclosure, B top panel) and the control samples (**(b)**, top panel): positive control (solid red enclosure), cut off (solid green enclosure), and negative control (solid blue enclosure). Refer to S7 for enlarged inset figure.

Following the successful demonstration of the efficacy of the MATLAB application, we proceeded to evaluate its efficiency to detect the extent of HRP-2 antibodies in buffer, serum, and blood (Fig. S3–S5, Supp. Information). The HRP-2 assay is a real-life assay used in malaria diagnosis [14]. The experiments were performed using the MABs technique and at room temperature without microwave heating and the results compared with those collected using an UVVis spectrophotometer. Fig. 1 shows the colorimetric response and the grayscale pixels for HRP-2 assay in blood performed using the MABs technique (total assay time < 20 min). From the optical absorption spectra shown in Fig. 1(a), all the samples in the concentration range 0.565–0.00565 mg/mL, exhibited absorbance of >0.4 compared to background (control experiments: positive, cut-off, and negative), which displayed absorbance of <0.1. We also observed the highest (~0.8) and the lowest (0.4) absorbance values for samples with concentrations 0.0113 mg/mL and 0.565 mg/mL, respectively (Fig. 1(a), bottom panel). Fig. 1(b) top panel shows the real color pictures for the enzymatic product for HRP-2 assay in blood.

The color is deep in sample wells (solid orange enclosure) compared to control experiments: +ve (solid red enclosure), cut-off (solid green enclosure) and -ve, (solid blue enclosure). When the picture of the sample was uploaded into our software and converted into grayscale, the pixel intensity for each well was computed, and the yield is as shown in Fig. 1(b), bottom panel. The grayscale pixel intensity was lowest (~183 pixels) for samples with a concentration of 0.565 mg/mL, and the grayscale pixel intensity increased gradually to the highest pixels of ~215 pixels, which was associated with 0.0565 mg/mL and 0.113 mg/mL before slightly reducing to ~ 210 pixels for 0.565 mg/ mL samples. Subsequently, the pixel intensity for 0.113 mg/mL, 0.0113 mg/mL and 0.00565 mg/mL were 205, 215 and 210 pixels, respectively. From our software, the LLOD for HRP-2 assay was undistinguishable because the +ve control: red bold horizontal lines generated higher normalized pixels (~1.1 pixels) compared to cut-off: green bold horizontal lines (~1.0 pixels) and -ve: blue bold horizontal lines (~0.94 pixels) control samples (Inset, Fig. 1(b), lower panel). However, under the UV-Vis analysis shown in Fig. 1(a) (bottom panel), we observed an LLOD of 0.00565 mg/ mL. It should be noted that the horizontal lines across each graph represents absorbances (in UV-vis analysis) and pixel values (in novel MATLAB software analysis) for control samples; red line (+ve control sample), green line (cut-off) and blue line (-ve control sample).

**Fig. 2:**
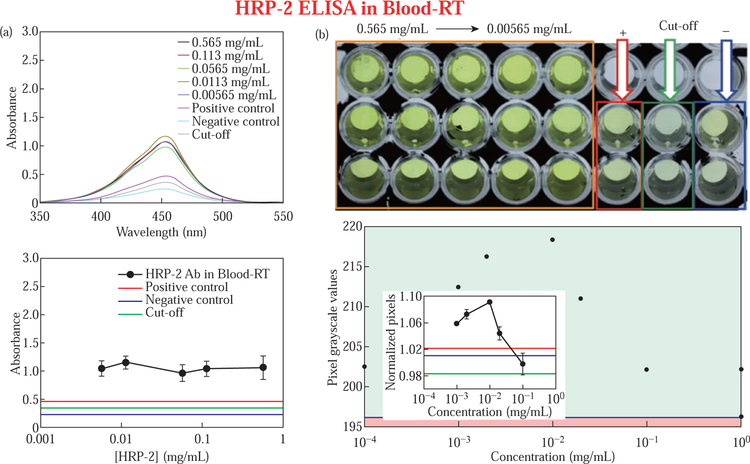
Colorimetric responses for HRP-2 assay in blood on modified ITO bioassay platform (A) at room temperature and grayscale pixel values computed using the new diagnostic software (B, bottom panel). The experimental samples (solid black enclosure, B top panel) and the control samples (B, top panel): positive control (solid red enclosure), cut off (solid green enclosure), and negative control (solid blue enclosure). Refer to S7 for enlarged inset figure.

Fig. 2 displays the results of a HRP-2 assay in blood conducted at room temperature without microwave heating as a control experiment. UV-vis analysis of the HRP-2 assay showed that the highest and lowest absorbance values were 1.1 and 0.9 for 0.0113 mg/mL and 0.565 mg/mL samples, respectively. Other samples yielded absorbance values as follows: 0.565 mg/mL (abs = 1.06), 0.00565 mg/mL (abs = 1.05) and 0.113 mg/mL (abs = 1.04). Subsequently, the control samples displayed the following absorbance values: positive (abs = 0.4638), cut off (abs = 0.3553) and negative (abs = 0.2386) (Fig. 2(a), bottom panel). The image is shown in Fig. 2(b) for the enzymatic product has the same organization and display as previously described in Fig. 1(b). However, the control samples in Fig. 2(b) developed slightly deeper color compared to those in Fig. 1(b) and this can be attributed to the long waiting periods at room temperature, which affords for more antigen-antibody complex formation. As a result, these complexes attach to the polyclonal antibodies bound to HRP enzyme and following the addition of a substrate, the enzyme breakdown the substrate to yield yellow color. The color produced is proportional to the amount of chromogen bound, which is directly proportional to the amount of antigen-antibody complexes. Therefore, Fig. 2(b), top-level (solid black line enclosure) depicts that the amount of antigen-antibody complexes formed in test samples are significantly predominant compared to those formed in control experiments. Fig. 2(b), bottom panel, demonstrates the output from our software in which the pixel intensity increased with a reduction in test sample concentration for the first three samples: 0.565 mg/mL (202 pixels), 0.113 mg/mL (210 pixels), and 0.0565 mg/mL (218 pixels). After the third sample, we observed a decline in pixel intensity, that is, 0.0113 mg/mL (216 pixels) 0.00565 mg/mL (212 pixels) and these reductions in pixel intensity can be attributed to the loss of pixels when the colored test sample picture is converted to grayscale format.

From the grayscale computation results for HRP-2 assay test sample pictures, we noted that the pixel output was not comparable with the real-color images of the enzymatic product in the wells. In this regard, we investigated whether pixel computation using the RGB color format can yield better and consistent results for HRP-2 test sample pictures. Since the colors for the enzymatic product for our HRP-2 assay is yellow, a construct of red, green, and blue proportions, we studied the effect of each color component in the three colors in the saturation of yellow color. Using random samples with an insignificant difference in saturation for the yellow color we performed RGB format pixel computation. Fig. S6 (Supp. Information) summaries the full RGB grid for the random samples taken on a 21-well bioassay plate using a 12 MP iPhone camera. Although the intensity of yellow color appears uniform in 21-wells, the distribution of intensity in the constituent colors (i.e., RGB) is apparent. The pixels values for green and red colors have an equal distribution (~250 pixels) in all the 21 wells. However, the pixel values for blue color are varied in most of the wells (Fig. S6B, Supp. Information). From these observations, it was evident that the blue color constituent (not green or red color component) determines the extent of brightness in the yellow color and as such, RGB format can be utilized for calibration in our novel software.

**Fig. 3:**
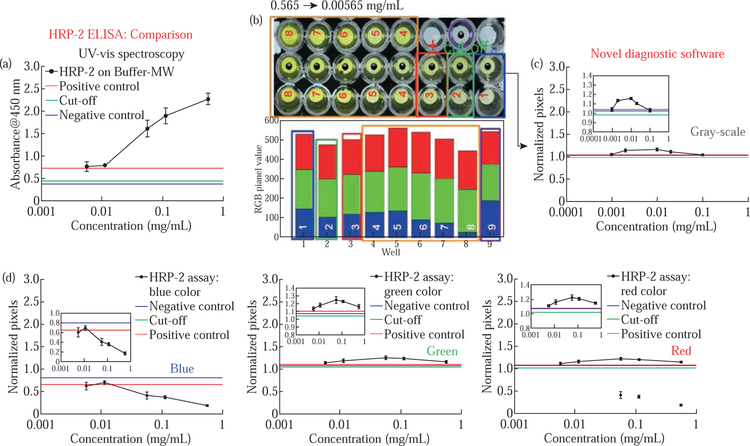
Colorimetric response for the HRP-2 assay (A), real-color pictures for the enzymatic product inside HTS wells (B) and their corresponding grayscale pixel intensities (C) and RGB channel pixel intensities (D). HRP-2 assay in buffer was completed using the MABs technique. Refer to S7 for enlarged inset figure.

To verify the observations mentioned above that blue color shows the widest variation in the brightness of yellow color developed as a result of colorimetric bioassays, we performed a real-life assay, HRP-2 bioassay, in the dynamic range 0.565–0.00565 mg/ mL using the MABs technique (Fig. 3). We note that the contents of HTS wells are colored enzymatic product from HRP-2 assay performed on ITO bioassay platforms and transferred into the HTS wells with a stop solution to end the enzymatic reaction. Well numbers represent different HRP-2 antibody concentrations, i.e., 8 = 0.565 mg/mL, 7 = 0.113 mg/mL, 6 = 0.0565 mg/mL, 5 = 0.0113 mg/mL, 4 = 0.00565 mg/mL, 3 = positive control, 2 = cut-off, 1 = negative control and 9 = blank sample (Fig. 3(b)). The absorbance values at 450 nm (Fig. 3(a)), grayscale and RGB pixel intensities (Fig. 3(c) and (d)) for the colored product were determined using UV-vis spectroscopy and our new MATLAB-based application, respectively. The optical absorbance spectra indicate that sample absorbance values increased with increase in the concentration for test samples (Fig. 3(a)). These observations implied that high concentrations of HRP-2 antibodies can readily be recognized and combined with the immobilized HRP-2 antigens yielding a higher number of antigen-antibody complexes.

Upon the addition of HRP-conjugate (detector antibody) and later to a colorimetric bioassay substrate (tetramethylbenzidine), the yellow color whose intensity (deep yellow = high absorbance, light yellow = low absorbance) varied depending on the concentration/number of antibody-antigen complexes developed. However, the output from the grayscale pixel computation for the HRP-2 assay image on Fig. 3(c), showed that the pixels of the first three samples (0.565 mg/mL, 0.113 mg/mL and 0.0565 mg/mL) increased linearly with a decrease in sample concentration and then the pixels reduced for the last two samples (0.0113 mg/mL and 0.00565 mg/ mL). These observations can be well explained using the image in Fig. 3(b) where wells 8, 7, and 6 which represent concentrations 0.565, 0.113 and 0.05656 mg/mL), display deep yellow color than the rest of the wells therefore after image conversion from color to grayscale, these wells (8, 7, and 6) appear darker relative to wells 5 and 4, hence the low grayscale pixels relation to wells 5 and 4. The grayscale pixels intensities for 0.0113 and 0.00565 mg/mL and control samples show a reduction in intensity, and this can be attributed to the loss of pixels during image conversion.

Conversely, the same picture image when subjected to RGB pixel computation generated interesting results. As shown in Fig. 3(d), the green and red components produced results similar in trend to grayscale pixel computation, that is, increase in pixel values with a decrease in the concentration for the first three samples. However, for the blue constituent, the pixel values consistently increase with a reduction in sample concentration (Fig. 3(d), Blue) and these results are accommodating with the picture image and the histogram of stacked RGB pixel values (Fig 3(b), bottom panel). When we compared the output of optical absorbance from UV-vis spectrophotometric analysis and blue pixel computation, the relationship for absorbance values and pixels of yellow sample color is of inverse proportionality (Figs. 3A vs. 3D, blue). Subsequently, we observed an LLOD of 0.00565 mg/mL for HRP-2 assay in buffer completed at room temperature without microwave heating and analyzed using UV-vis and RGB pixel computation (blue constituent).

**Fig. 4:**
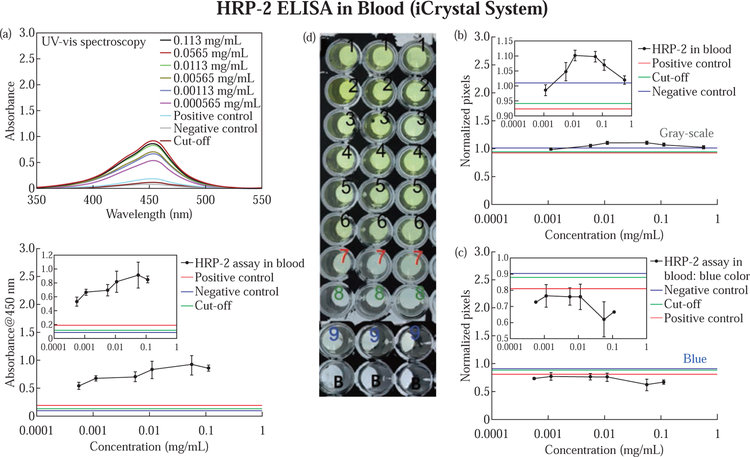
Colorimetric response for HRP-2 assay in blood completed using the MABs technique (A) and grayscale (C) and blue pixel computation (C) for the sample picture image (D) representing test samples (1–6), controls (7–9) and blank experiments. Legend: 1= 0.113, 2 = 0.0565, 3 = 0.0113, 4 = 0.00565, 5 = 0.00113, 6 = 0.000565 mg/mL. Controls: 7 = positive control, 8 = cut-off, 9 = negative control and B = blank sample. Refer to S7 for enlarged inset figure.

Fig. 4 displays the colorimetric response obtained using UV-vis spectroscopy, grayscale, and RGB (blue component) pixel computation for HRP-2 assay in blood completed using the MABs technique. The optical absorbance values are shown in Fig. 4(a), upper and lower panels, indicate the highest and lowest absorbance values of ~1.0 and ~0.5 in samples 0.0565 mg/mL and 0.000565 mg/mL, respectively. Other HRP-2 assay test samples produced the following absorbance values: 0.113 mg/mL (Abs = 0.85), 0.113 mg/mL (Abs = 0.82), 0.00565 mg/mL (Abs ~ 0.7), and 0.00113 mg/mL (Abs = 0.67).

We observed absorbance values 0.19, 0.08 and 0.12 for positive, cutoff, and negative control samples, respectively and from these results, we detected an LLOD of 0.000565 mg/mL for HRP-2 antibodies. Subsequently, we determined the grayscale pixels for the enzymatic product for HRP-2 assay in blood (Fig. 4(b)). From these results, we observed that the pixels values increased with a decrease in test sample concentration for the first four sample trials (i.e., 0.565 mg/mL (pixels = 1.01), 0.113 mg/mL (pixels = 1.06), 0.0565 mg/mL (pixels = 1.09), and 0.0113 mg/mL (pixels = 1.1), but the grayscale pixels reduced with a reduction in specimen concentration in the last two samples (0.00565 mg/mL (pixels = 1.0), and 0.00113 mg/mL (pixels = 0.98) (Fig. 4(b), Inset). The control samples exhibited the lower greyscale pixel values compared to the test results. That is, positive control (pixels = 0.92), cut-off (pixels = 0.94 and negative control (pixels = 1.01). However, the pixel trend was different when we subjected the samples images into RGB (blue component) pixel computation. The blue pixel values for the HRP-2 assay images consistently increased with a decrease in sample concentration except for the first (0.113 mg/mL) and last (0.000565 mg/mL) sample sets which showed a slightly high (0.66 pixels vs. 0.62 pixels) and lower (0.73 pixels vs. 0.77 pixels) blue pixels values compared to those in the next and previous sample neighbors, respectively (Fig. 4(c)). The control samples, positive, cut-off and negative controls, showed higher blue pixel values (positive control = 0.81, negative control = 0.90 and cut-off = 0.88) than HRP-2 antibody samples. In both RGB – blue pixel computation and Uvvis spectrometric analysis, we witnessed LLOD of 0.000565 mg/mL for HRP-2 assay in blood completed using the MABs technique (Fig. 4A and C).

#### Application of MATLAB-based Software in MC-LR Sensing

**Fig. 5:**
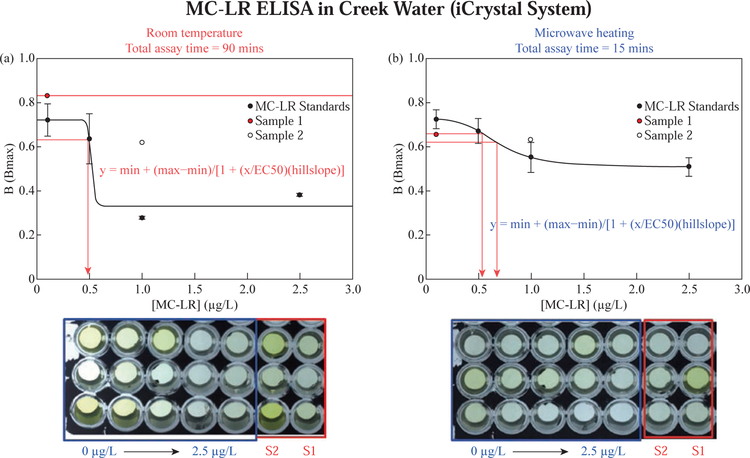
Colorimetric responses for MC-LR assay in buffer and MSU creek water for MC-LR standards and unknown samples (S1 and S2) performed on ITO bioassay platforms at room temperature without microwave heating (A) and using the MABs technique (B). Experimental products were analyzed using UV-spectrophotometer.

Fig. 5 shows the colorimetric response for MCLR assay in creek water for MC-LR standards and unknown samples (labeled S1 and S2) performed on ITO bioassay platforms using the MABs technique and at room temperature without microwave heating (total assay time = 90 mins). The absorbance of the colored enzymatic product was analyzed using UV-vis spectrophotometer. Fig. 5(a) displays a standard curve for normalized absorbance values for MC-LR assay standard samples (concentrations: 0 mg/L, 0.1 mg/L, 0.5 mg/L, 1.0 mg/L and 2.5 mg/L) completed at room temperature (total assay time = 90 mins). Using the standard curve of [y = min+(max-min)/ (1+(x/EC50)^(hillslope))] the estimated concentrations for the creek water spiked with MC-LR toxin were determined. The concentration of sample 1 (filled red circle) was undefined because when a trace line was drawn across the graph, it never intersected with the standard curve. The estimated concentration of sample 2 (empty white circle) was ~ 0.5 mg/L. Fig. 5(b) shows results for MC-LR assay completed under microwave heating (total assay time = 15 mins) with the estimated concentration for sample 1 and sample 2 being ~ 0.5 and ~ 0.7 mg/L, respectively. The difference in the results can be attributed to the MABs technique which is well detailed in the previously published work [12, 13, 15, 16].

**Fig. 6:**
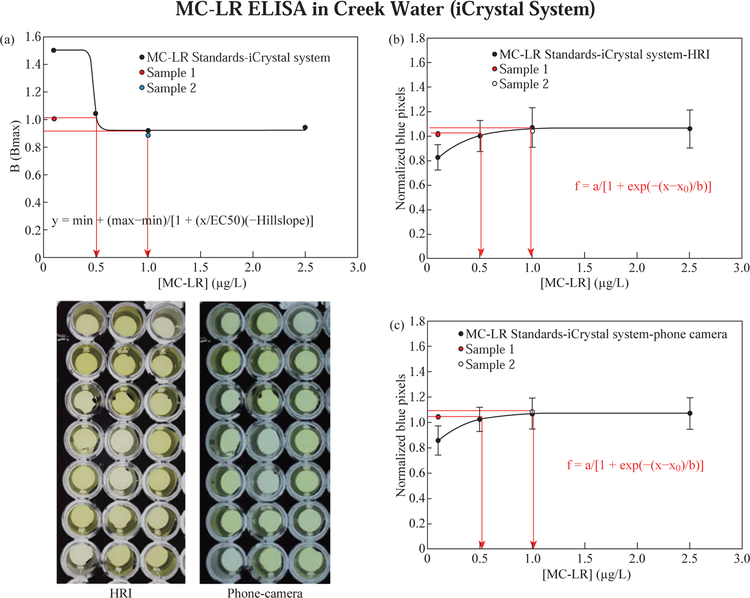
Colorimetric responses and pixel computation for MC-LR assay in buffer and MSU creek water of MC-LR standards and samples (S1 and S2) performed on HTS wells using the MABs technique using our MATLAB application. The assay pictures were obtained using the high-resolution camera and smartphone camera.

To evaluate whether the type of camera influenced the outcome in bioassays, we took pictures of the enzymatic colored product for MC-LR assay using an 12 MP iPhone camera and a 80 MP high resolution camera (Phase One) and compared the MATLAB computed pixels (blue pixels) with absorbance values obtained using an UV-vis spectrophotometer (Fig. 6). Fig. 6(a) shows the absorbance results for MCLR assay using UV-vis analysis. The standard curve shows that the low concentration of MC-LR displayed higher absorbance values compared to higher MC-LR concentrations leading to a reverse sigmoid standard curve. This observation can be explained that given the MC-LR assay is a competitive assay, low concentration of MC-LR toxin leads to the development a deep color at the end of the assay which translates into higher absorbance values and vice versa. From the UV-vis analysis, the concentration for the unknown sample S1 was estimated to be 0.5 μg/L while the concentration for unknown sample S2 was 1.0 μg/L (Fig. 6(a)).

Fig. 6(b) and (c) show the normalized blue pixels (range ~0.8 to ~1.07 pixels) for MC-LR assay images obtained using an 80 MP high resolution and a 12 MP iPhone camera, respectively. In both figures, the pixel values increase with increase MC-LR concentration for the first three standard samples and then levels off. At low (<0.1 μg/L) MC-LR concentration, the color of the enzymatic product was darker, which translated in to fewer blue pixels (~0.8 pixels). At higher MC-LR toxin concentration (> 1.0 μg/L), a less intense yellow color develops which gives rise to high blue pixel values (~> 0.9 pixels). In the determination of the concentration for the unknown samples, S1 and S2, images were taken with HRI (80 MP) and iPhone camera (12 MP) yielded the same concentration for unknown samples: that is, sample S1 = 0.5 μg/L and S2 = 1.0 μg/L. From these results, we were convinced that the resolution of the camera does not influence the MC-LR assay results.

#### Conclusions

In this study, we have successfully demonstrared the use of a MATLAB-based application for the analysis of colorimetric signal generated from a model protein bioassay for b-BSA, where our MATLAB-based application computed pixel intensities in the range 0–255 of the colored enzymatic product in grayscale and RGB formats. Using a model bioassay model system of the b-BSA assay and grayscale pixel computation, we observed LLODs of [b-BSA] =10^−8^ M and [b-BSA] = 10^−7^ M for b-BSA bioassay completed on silvered and blank PMMA bioassay platforms using the MABs technique, respectively. HRP-2 in buffer and blood completed at room temperature yielded dense color (Abs >2.0 and >1.0, respectively) as compared to HRP-2 assay in serum (abs > 0.5). LLODs of 0.0113 mg/mL and 0.00565 mg/mL were observed for HRP-2 assays in buffer and serum and, blood, respectively. Using the MABs technique, HRP-2 assay in buffer and blood yielded absorbance values >1.0 and <1.0 and LLODs of 0.0113 mg/mL and 0.00565 mg/mL, respectively. HRP-2 assay in serum produced absorbance of < 0.5 and LLOD of 0.00565 mg/mL. HRP-2 assay test sample images analyzed using grayscale pixel intensity computation showed inferior LLODs of 0.113 mg/mL as compared to the results of the same samples under UV-vis analysis which displayed LLODs of 0.00565 mg/mL. Our MATLAB-based application was efficient in HRP-2 assay samples, which developed deep yellow color compared to those that appeared lighter, and this was attributed to the loss of pixels during the conversion of colored sample images to grayscale picture format. Due to this limitation presented by the grayscale format, RGB (red, green, and blue) color scheme was instead used to calculate the pixels values for the yellow colored enzymatic product. Subsequently, the green and red pixels did not vary significantly from each other implying that blue pixels regulated the luminosity of the yellow color. In that regard, the blue color channel was used in real-life assays (i.e., HRP-2 and MC-LR assay) experiments performed using the MABs technique and at room temperature without microwave heating as control experiments. A blue constituent of the RGB format yielded LLOD of 0.000565 mg/mL for HRP-2 assay test sample images completed using the MABs technique, which were similar to LLOD produced for the same samples under UV-Vis spectrometric analysis (LLOD = 0.00565 mg/mL). Notably, the type of camera (in terms of pixels) does not affect the outcome of the bioassay results provided pixel normalization be performed. Our MATLAB-based application has demonstrated remarkable sensitivity, shown affordability and portability. However, it is only available to only licensed users. In that regard, we are currently implementing the same algorithm in two mobile platforms: iOS and Android and report these results in due course.

## Supplementary Material

S1-S7
