# Supplementary material for: Rapid Sensing of Biological and Environmental Analytes Using Microwave-Accelerated Bioassays and a MATLAB Application
Source: Nano Biomed Eng. Author manuscript; Available in PMC 2019 Sep 27. (PMC6764453; doi:10.5101/nbe.v11i2.p111-123)
Supplement: S1-S7 [file NIHMS1038170-supplement-S1-S7.docx]

**Rapid Sensing of Biological and Environmental Analytes using Microwave-Accelerated Bioassays and a MATLAB Application**

Enock Bonyi, ^1^ Edward N. Constance, ^1^ Zeenat Kukoyi, ^1^ Sanjeeda Jafar, ^2^ and Kadir Aslan*, ^1^

^1^Department of Civil Engineering, Morgan State University, 1700 East Cold Spring Lane, Baltimore, Maryland 21251

^2^Department of Biology, Morgan State University, 1700 East Cold Spring Lane, Baltimore, Maryland 21251

*Corresponding Author: [Kadir.Aslan@morgan.edu](mailto:Kadir.Aslan@morgan.edu)


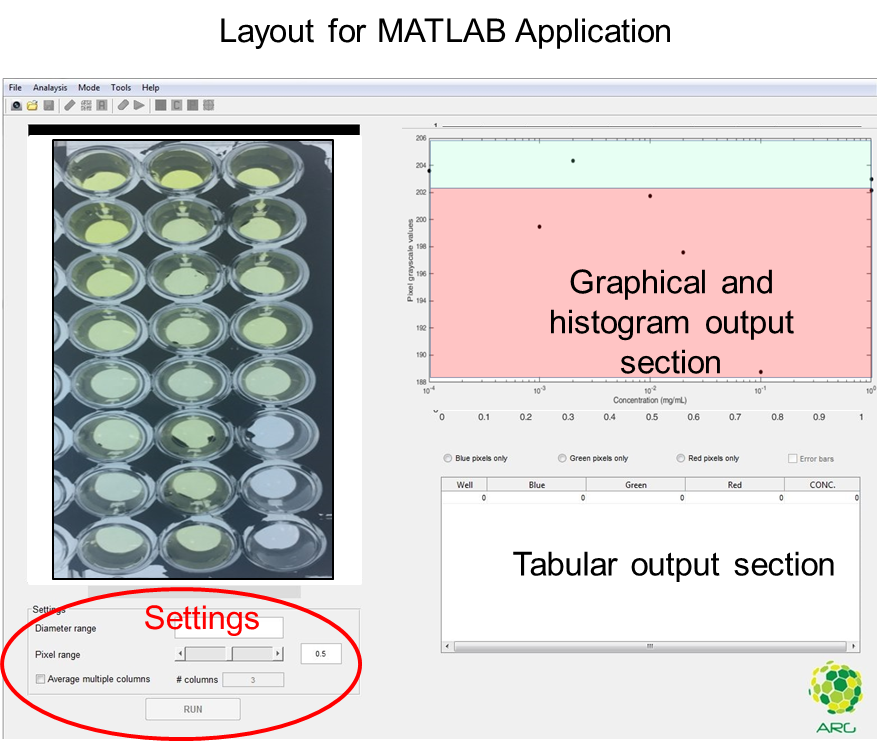


**S1:** Layout for the MATLAB application with four sections: Image upload, Graphical and histogram output, Tabular output and Settings sections.


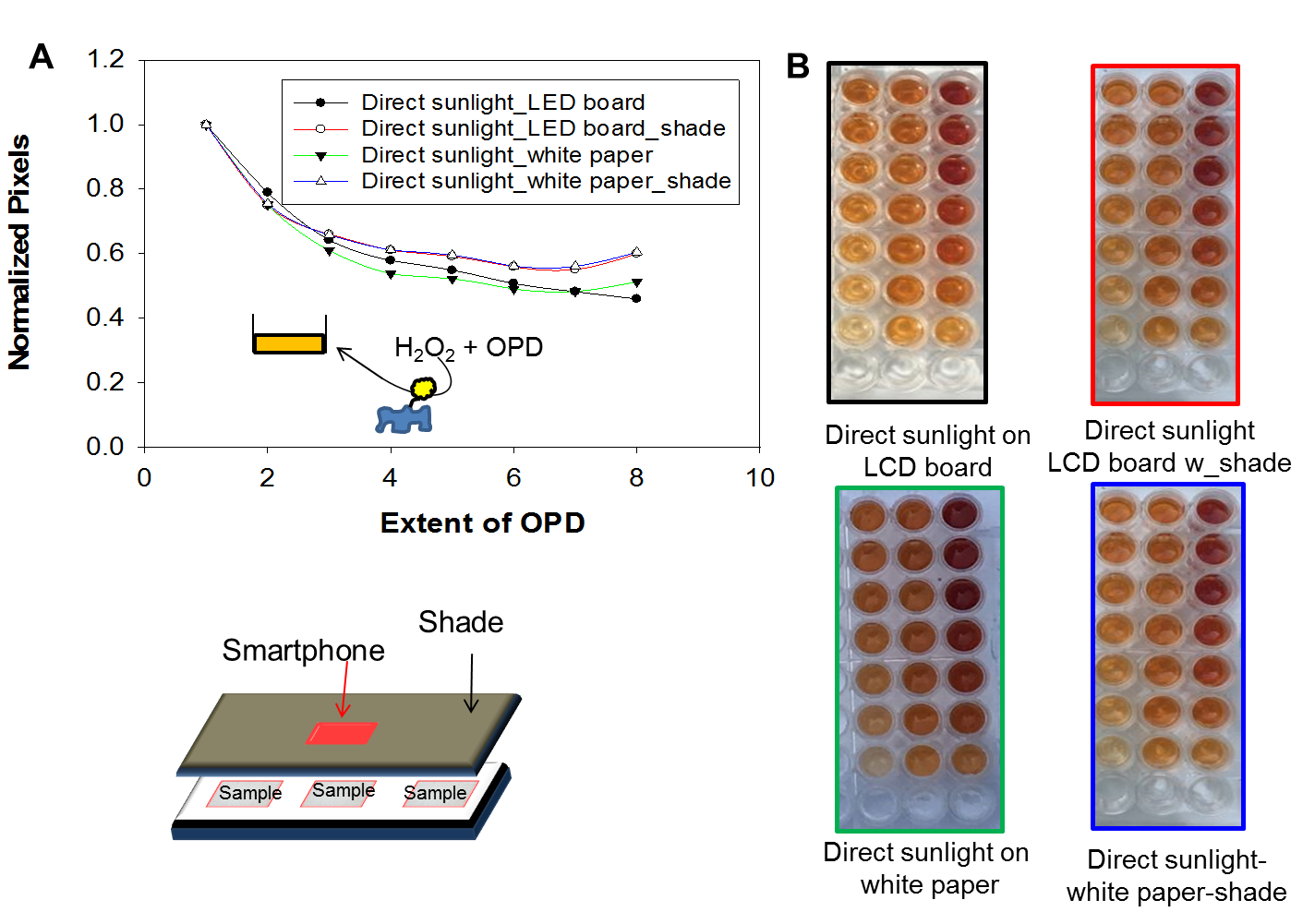


**S2** Normalized pixels for enzymatic product o-phenylenediamine dihydrochloride (OPD) generated by varying volumes of OPD while maintaining the enzyme, streptavidin-horseradish peroxidase (Strep-HRP) constant (left panel) carried out in direct sunlight and under a shade. Real color images of OPD product samples exposed to direct sunlight (black and green solid line enclosure) and under a shade (red and blue solid line enclosure).


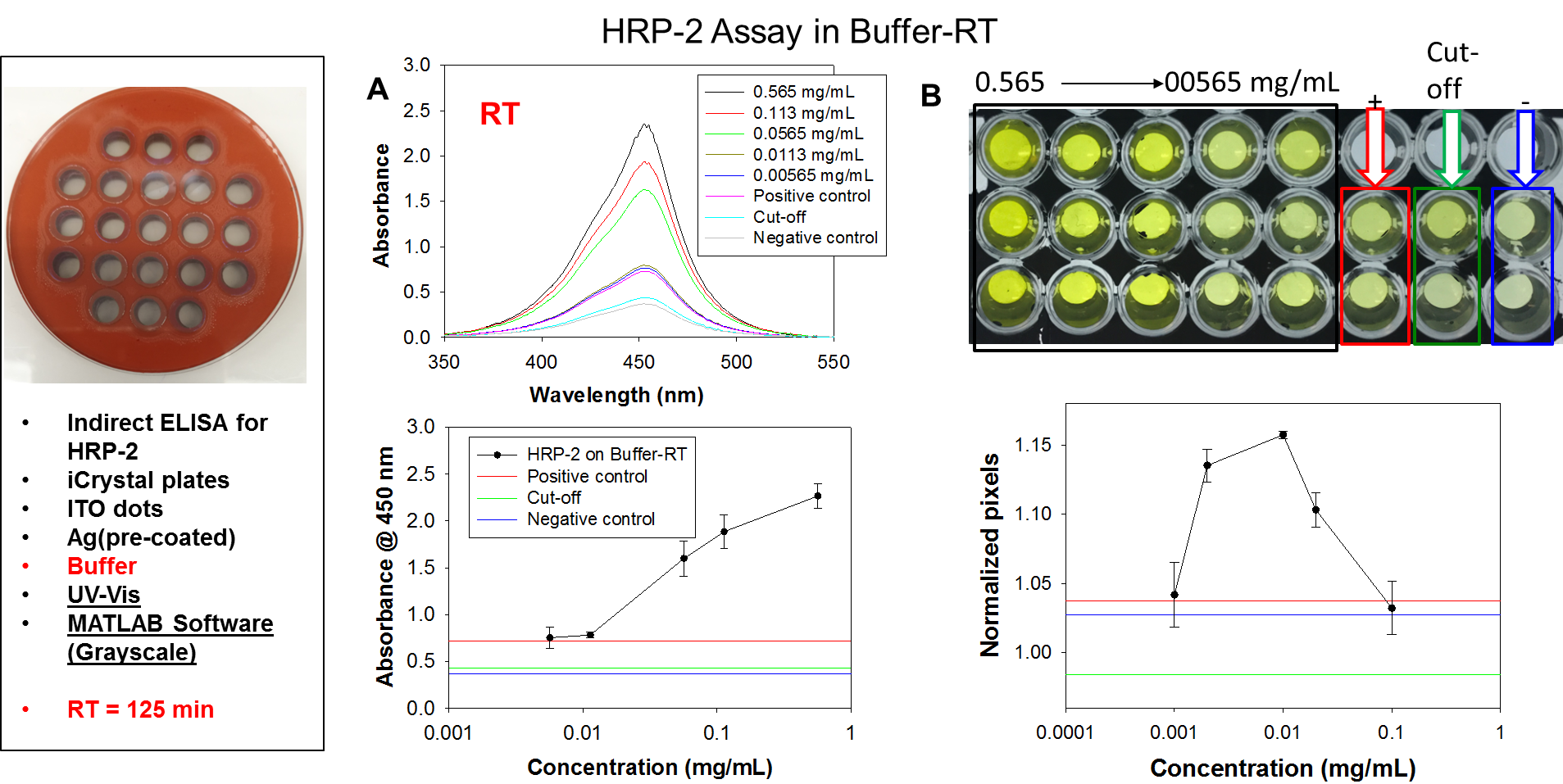


**S3:** Colorimetric response for HRP-2 assay in buffer on modified ITO platform (A) at room temperature and grayscale pixel values computed using the novel diagnostic software (B, bottom panel). The experimental samples (black solid enclosure, B top panel) and the control samples (B, top panel): positive control (red solid enclosure), cut off (green solid enclosure), and negative control (blue solid enclosure). The substrate volume was increased 3-fold.


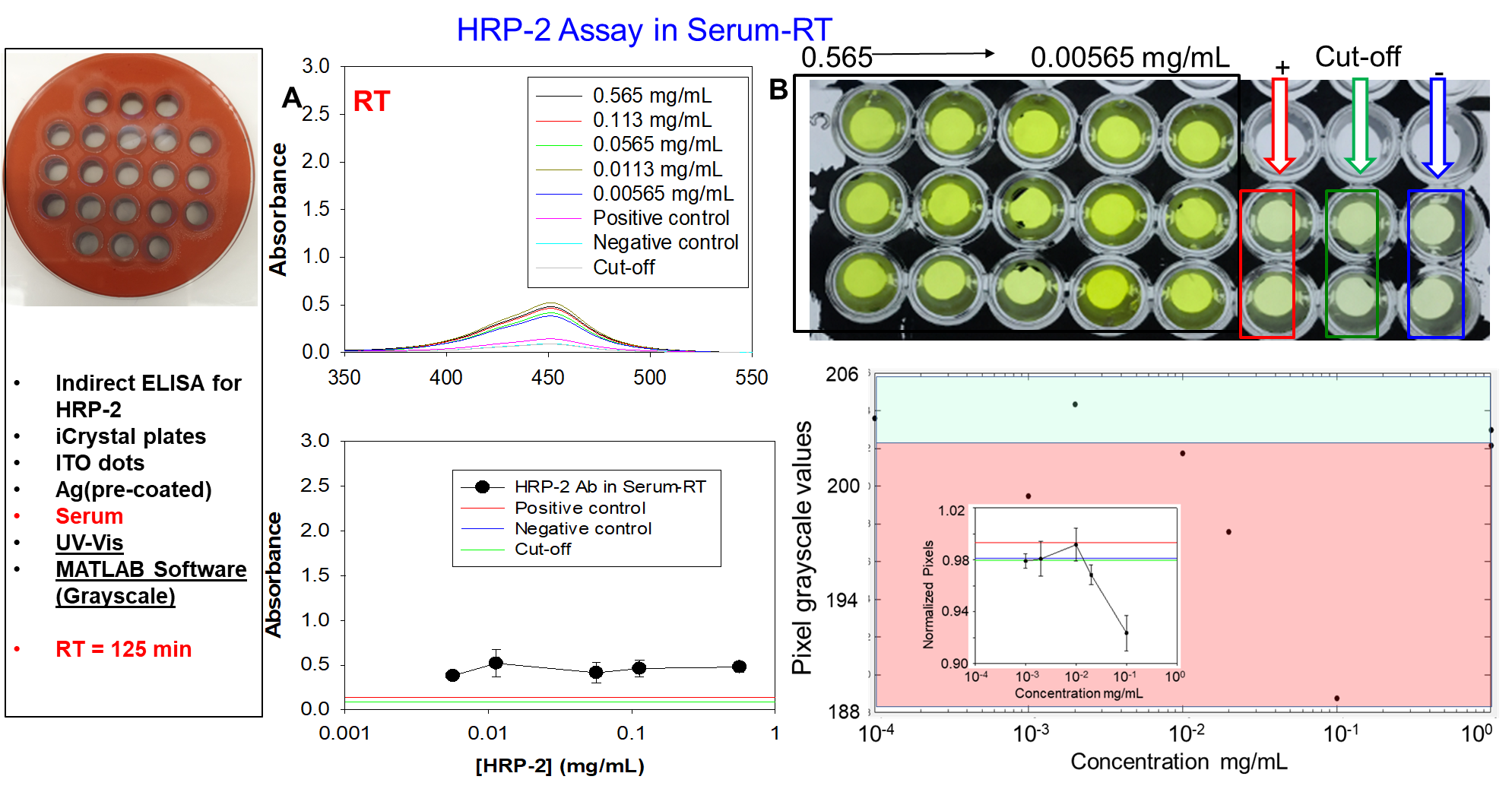


**S4:** Colorimetric response for HRP-2 assay in serum on modified ITO platform (A) at room temperature and grayscale pixel values computed using the novel diagnostic software (B, bottom panel). The experimental samples (black solid enclosure, B top panel) and the control samples (B, top panel): positive control (red solid enclosure), cut off (green solid enclosure), and negative control (blue solid enclosure). The substrate volume was increased 3-fold.


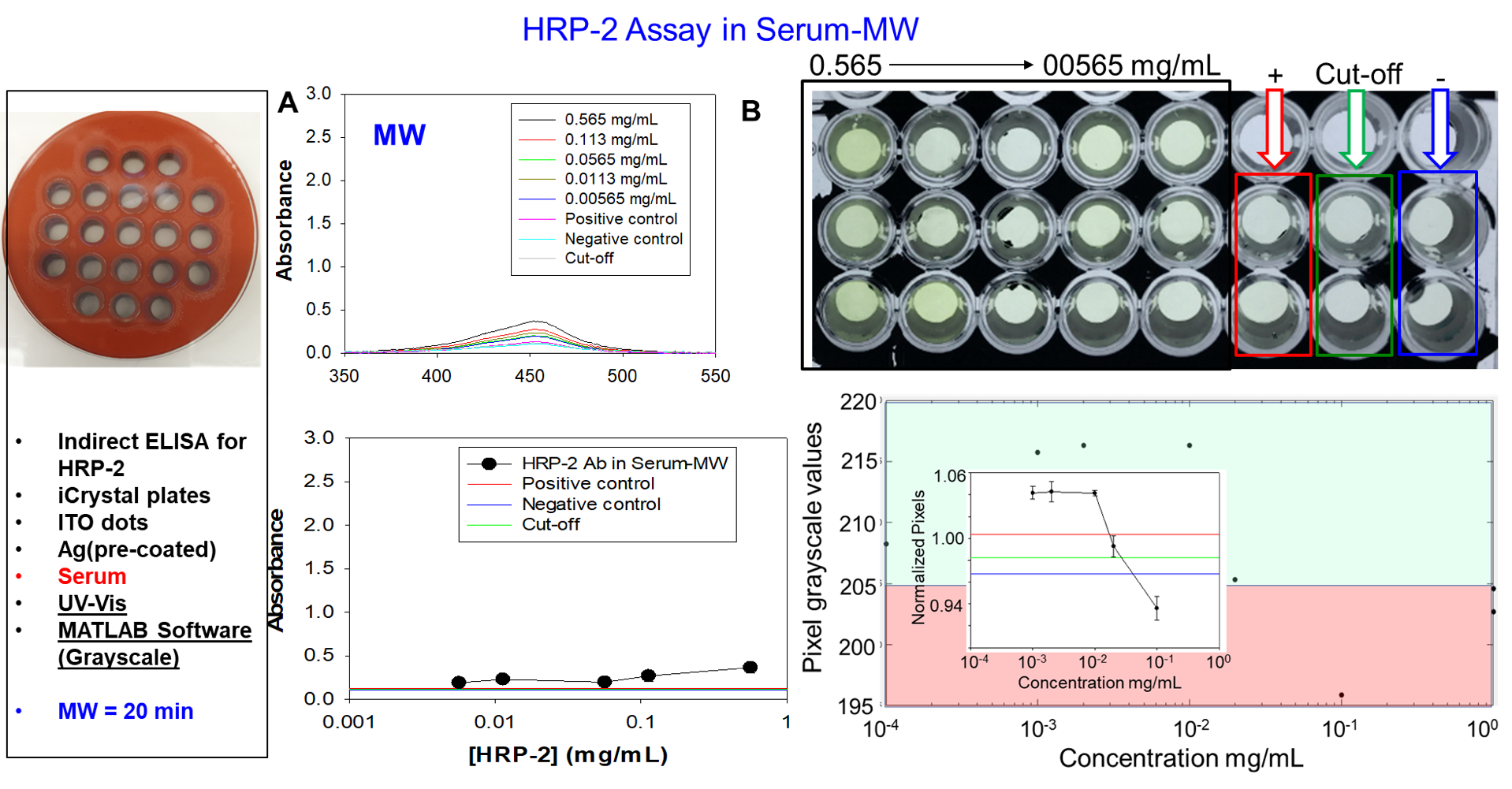


**S5:** Colorimetric response for HRP-2 assay in serum on modified ITO platform (A) under low power microwave heating and grayscale pixel values computed using the novel diagnostic software (B, bottom panel). The experimental samples (black solid enclosure, B top panel) and the control samples (B, top panel): positive control (red solid enclosure), cut off (green solid enclosure), and negative control (blue solid enclosure). The substrate volume was increased 3-fold.


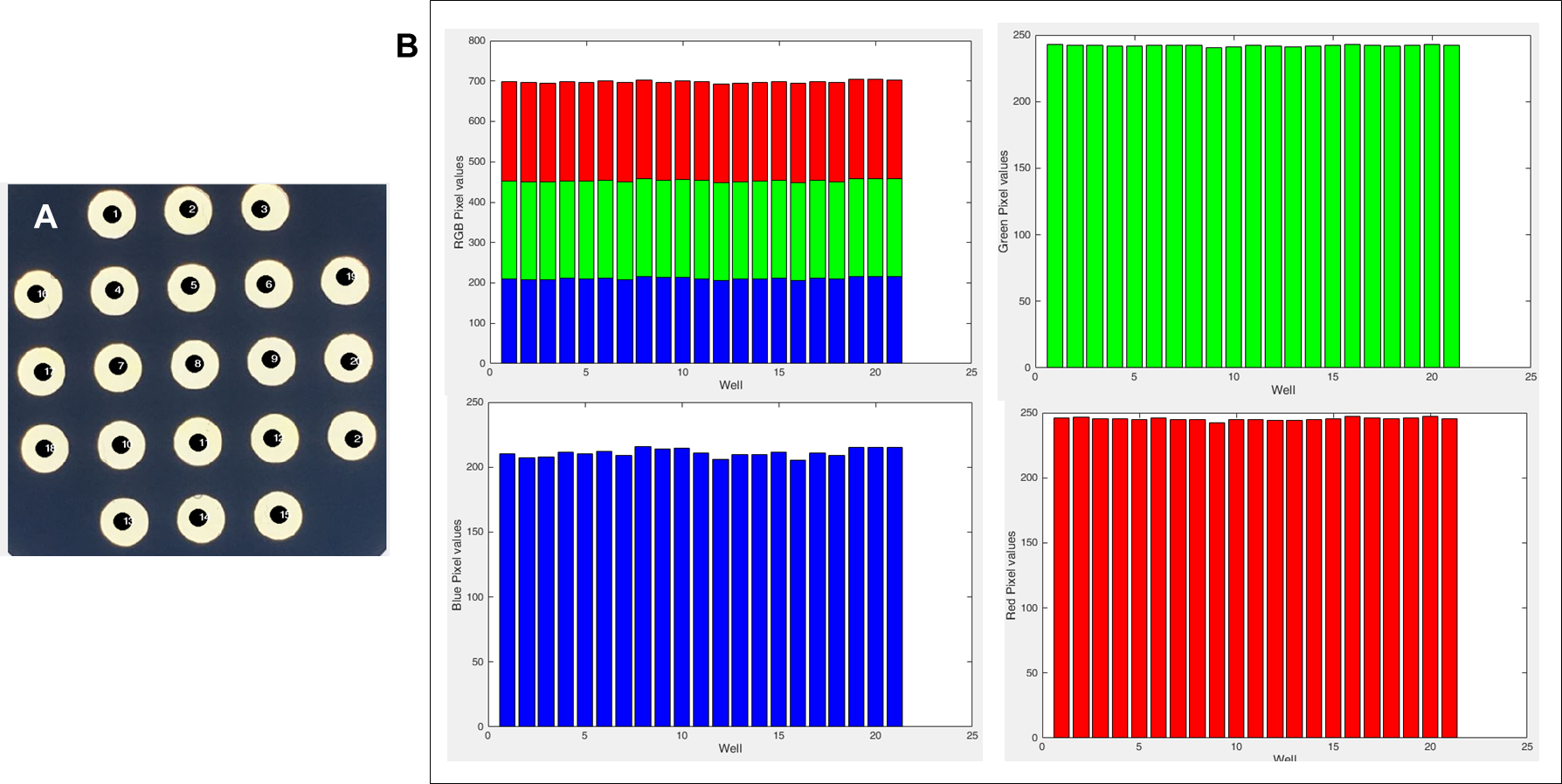


**S6:** RGB format pixel output for random test samples on a 21-well iCrystal Plate (A) and individual pixel intensity values for Green, Blue and Red colors (B).


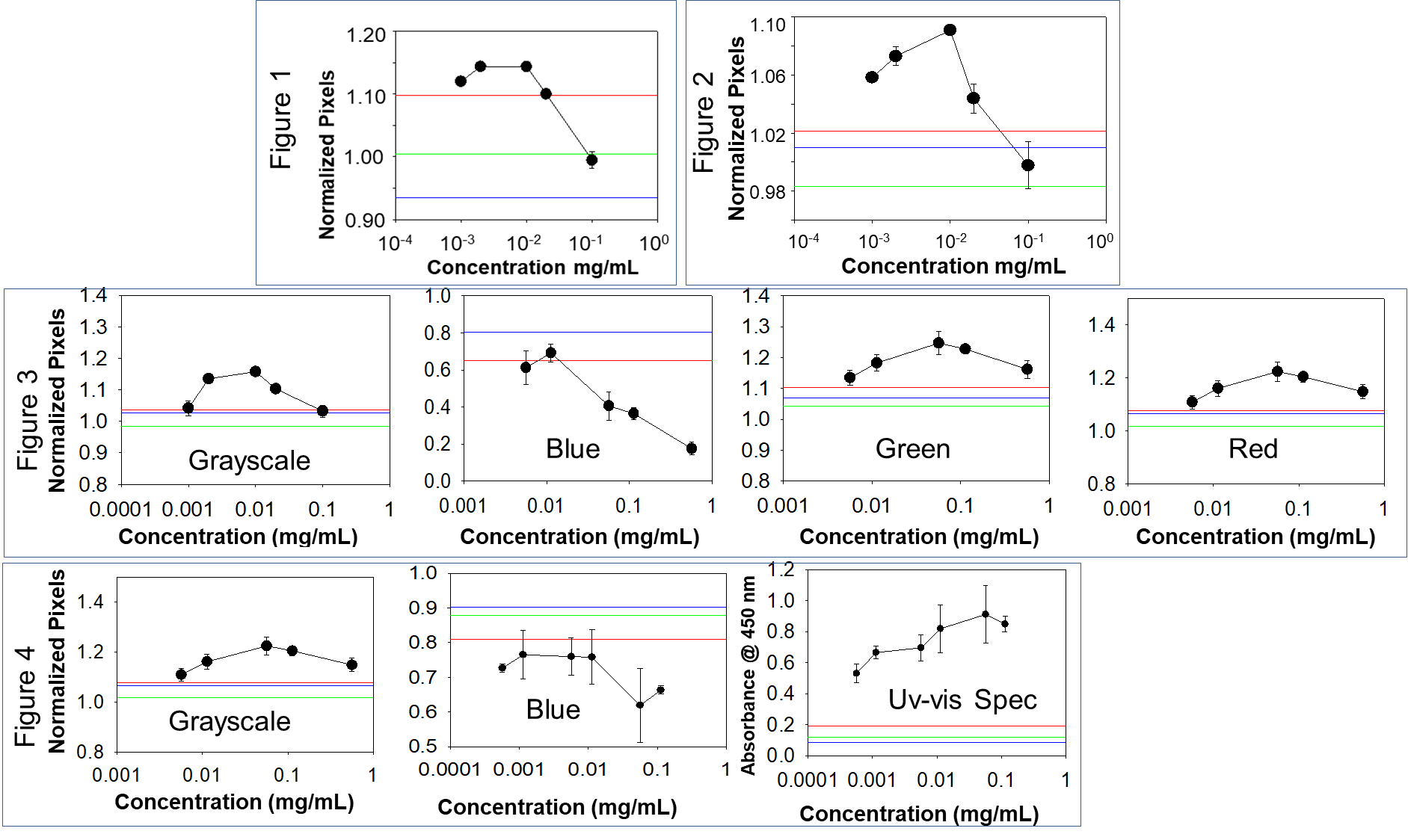


**S7:** Insets for Figs.1, 2, 3 and 4.
